# Heterogeneous Organocatalysts for Light-Driven Reactions in Continuous Flow

**DOI:** 10.3390/molecules29102166

**Published:** 2024-05-07

**Authors:** Graziano Di Carmine, Carmine D’Agostino, Olga Bortolini, Lorenzo Poletti, Carmela De Risi, Daniele Ragno, Alessandro Massi

**Affiliations:** 1 Department of Environmental and Prevention Sciences, University of Ferrara, Via Luigi Borsari 46, 44121 Ferrara, Italy; brl@unife.it; 2 Department of Chemical Engineering, University of Manchester, Oxford Road, Manchester M13 9PL, UK; carmine.dagostino@manchester.ac.uk; 3 Department of Civil, Chemical, Environmental, and Materials Engineering, Alma Mater Studiorum—University of Bologna, Via Terracini 28, 40131 Bologna, Italy; 4 Department of Chemical, Pharmaceutical and Agricultural Sciences, The University of Ferrara, Via Luigi Borsari 46, 44121 Ferrara, Italy; lorenzo.poletti@unife.it (L.P.); drc@unife.it (C.D.R.); daniele.ragno@unife.it (D.R.); alessandro.massi@unife.it (A.M.)

**Keywords:** photocatalysis, flow chemistry, heterogeneous catalysis, synthetic methodologies, green chemistry

## Abstract

Within the realm of organic synthesis, photocatalysis has blossomed since the beginning of the last decade. A plethora of classical reactivities, such as selective oxidation of alcohol and amines, redox radical formation of reactive species in situ, and indirect activation of an organic substrate for cycloaddition by EnT, have been revised in a milder and more sustainable fashion via photocatalysis. However, even though the spark of creativity leads scientists to explore new reactions and reactivities, the urgency of replacing the toxic and critical metals that are involved as catalysts has encouraged chemists to find alternatives in the branch of science called organocatalysis. Unfortunately, replacing metal catalysts with organic analogues can be too expensive sometimes; however, this drawback can be solved by the reutilization of the catalyst if it is heterogeneous. The aim of this review is to present the recent works in the field of heterogeneous photocatalysis, applied to organic synthesis, enabled by continuous flow. In detail, among the heterogeneous catalysts, g-CN, polymeric photoactive materials, and supported molecular catalysts have been discussed within their specific sections, rather than focusing on the types of reactions.

## 1. Introduction

Discovering new processes or reactivity to access unknown compounds is as important as redesigning previous ones [1]. Making a process more sustainable than an already existing one involves replacing any toxic reactant and auxiliary with a greener one [2,3,4]; furthermore, these green alternatives, preferably, should come from renewable sources and the life cycle assessment (LCA) ought to provide positive responses, preferring the reuse and recycling of the catalyst and auxiliary rather than disposing of them [5,6]. The twelve principles of green chemistry, introduced by Paul Anastas and John Warner in 1998, have been a leading light during these last 20 years for both organic chemists and industrial chemists [7]. The ninth principle, “catalysis”, focuses on the urgency to replace stoichiometric reagents with catalysts, in order to avoid waste coming from co-products with high molecular weight. Interestingly, other points are strongly linked to the ninth one, making catalysis an important task for more sustainable chemistry. For example, in the first principle, the authors point out the importance of “prevention”, where preventing waste is preferable to treating it. Even in the second principle, “Atom Economy”, catalysis seems to be a valuable ally for the reason explained above. In principle number eight, “reduce derivatives”, certain kinds of catalysts can address the issue [7]. The use of expensive and time-consuming chiral auxiliaries is avoided by employing chiral catalysts that can drive the reaction toward target compounds with high optical purity [8]. In a few words, “catalysis” is “sustainable”, but is it possible to make it even more sustainable [9]?

**Figure 1:**
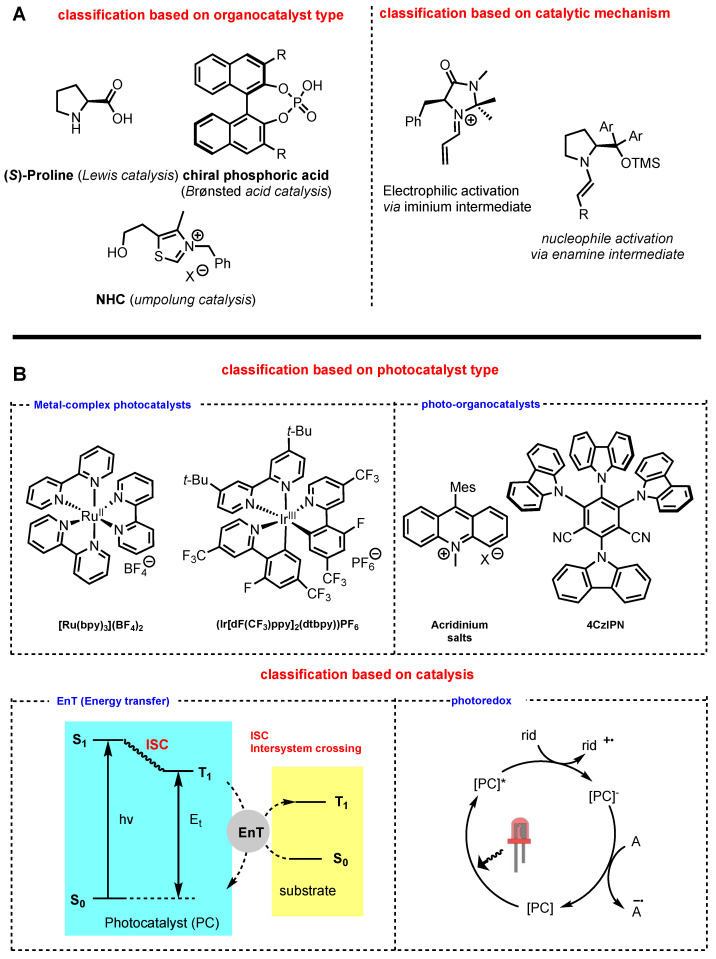
An example of catalysis classification in terms of the catalyst type or catalysis mode: (**A**) organocatalysis and (**B**) photocatalysis. * means excited state.

In order to answer this question, some features of catalysis, and of catalysts, have to be taken into account. First of all, how the catalyst is made needs to be considered: organocatalysts [10], abundant metal complexes, and biocatalysts are preferable to critical raw metals that are rare on the earth’s crust and require a huge amount of energy for extraction and separation [11,12,13]. Furthermore, heterogenous catalysis is welcomed and preferable to its homogeneous counterpart, making the purification process easier through mechanical separation (filtration, centrifugation etc.) and the possible reuse of the catalyst in further reactions [14,15,16,17]. The classification of catalysis is related to both the catalyst’s structure and function. When a catalyst is a small organic molecule, it belongs to the class of organocatalysts; however, its role can cover different reactivities, which depends on the activation mode and how the catalyst interacts with the reagent/reactant. For example, proline and proline-like catalysts promote the formation of new C–C bonds by means of either enamine or imine catalyst–substrate adducts (Figure 1A) [18,19]. In addition, N-heterocyclic carbenes (NHCs) are able to mediate a number of transformations through a typical type of umpolung (polarity reversal) reactivity. [20] Within the realm of catalysis, photocatalysis can be described as an activation mode (catalyst function) in which the catalyst, in its photo-activated form, interacts with the substrate; however, it can belong to different classes according to its structure, such as organocatalysts or metal catalysts. The exploitation of photochemistry, within the realm of catalysis, represents a valuable tactic to meet the demand for more sustainable catalysis. In terms of energy consumption, photochemistry is preferable to heat-promoted processes because reactions are activated by photons, a renewable source of energy, which comes from the sun and does not generate any kind of waste. In organic synthesis, photocatalysis is exploited mainly through either redox or energy transfer (EnT) mechanisms (Figure 1B) [21,22,23].

**Figure 2:**
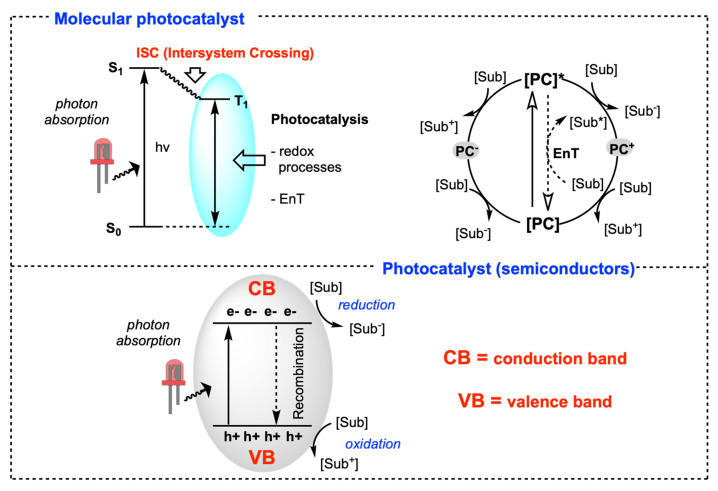
Simplified mechanisms, which involve molecular photocatalysts and semiconductors. * means excited state.

After the absorption of a photon by the catalyst, an electron is promoted to the next energetic level. The fate of this excitation is different from that of molecular photocatalysts in homogeneous conditions and in regard to semiconductors, as will be discussed in detail later in this review (Figure 2) [24].

**Figure 3:**
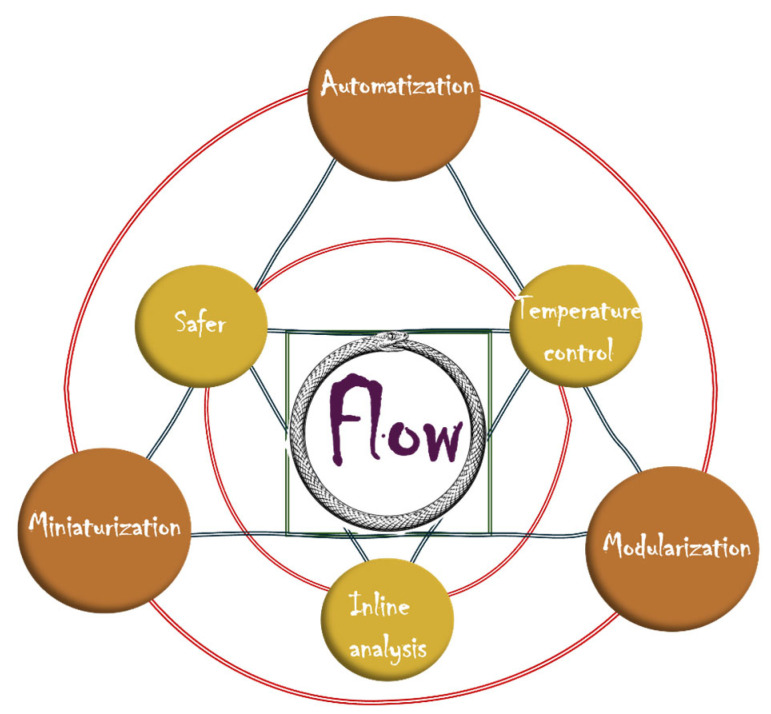
The image shows some advantages of continuous flow reactions compared to batch ones.

Semiconductors are heterogenous materials in which the reaction takes place at the interface of the solid, and the reagent is dissolved in liquid media; this feature makes this kind of photocatalyst reusable after mechanical separation from the reaction medium at the end of reaction and can be implemented in a continuous flow reactor [25]. However, it is important to point out that molecular photocatalysts can be immobilized onto an inert, insoluble support, in order to make them heterogenous as well; some other aspects have to be taken into account, however [26]. Continuous flow reactors and microfluidic technology have gained interest in the field of synthetic chemistry in the last two decades, with researchers aiming to enhance the possibility of performing chemical reactions with small to large-scale dimensions, providing total control over key reaction parameters [27,28,29,30]. Performing reactions in a continuous flow regime has some advantages: in general, the better temperature control and the lower reaction volume allow runaway reactions to be performed safely, accommodating higher pressures without risk, and offering the possibility to design a configuration with inline analysis as well (Figure 3) [31,32,33].

Optimal selectivity can be achieved through efficient heat transfer and mixing, whereas fast optimization of the reaction parameters and facile scale-up can be achieved by automatization and modular set-ups, respectively [34]. In particular, the modular design allows systematic scaling of the reactions by increasing the modules within parallel reactors through the so-called numbering-up assembly; in particular, this configuration is exploited for photoreactions in flow, due to the easy miniaturization of the reactors [35]. The typical architecture of microfluidic continuous flow reactors increases the surface area to volume ratio when compared with standard batch reactors, thanks to their small channel dimensions, providing an efficient and accurate level of illumination, which is particularly important for light-driven reactions. Aiming to provide a comprehensive review on the recent advances in light-driven reactions performed in continuous flow in the field of heterogeneous organocatalysis, herein we report on the works disclosed since 2020 as an update to previous reviews on this topic [36,37]. In order to best select and critically read the papers presented, a few pieces of background information should be reported in this regard.

## 2. General Features of Heterogeneous and Heterogenized Photocatalysts

**Figure 4:**
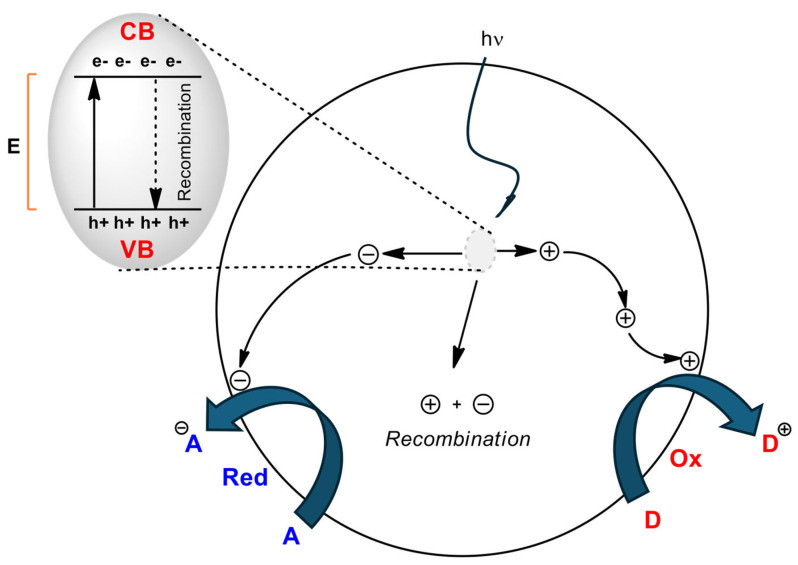
Mechanism of redox transformations mediated by generic semiconductors.

Heterogeneous photocatalysts that exhibit semiconductor behaviors have a working mechanism that differs from molecular catalysts. Basically, the effectiveness of a heterogeneous catalyst is greatly influenced by its morphology, since a heterogeneous catalyst with a large surface area is frequently associated with a greater number of surface active sites; however, for a heterogeneous photocatalyst, its morphology can drastically affect charge recombination events, thus some aspects should be addressed in this regard. Figure 4 depicts the first general step involved in photocatalysis mediated by semiconductors; the energy bands are delocalized throughout the crystal lattice of the semiconductor and the highest band (VB, valence band) is completely full, whereas the next band (CB, conduction band) is empty and separated from the VB by an energy difference (E_g_), which determines the minimum energy necessary for optical excitation. After the absorption of a photon, an electron from the VB is promoted to the CB; thus, a hole (h^+^) and an energetic electron (e^−^) are produced. This couple is named the electron/hole pair and is involved in both substrate reduction and oxidation. The components (h^+^/e^−^) are held together by an electrostatic interaction and the pair itself can be treated as a single neutral quasiparticle, named an exciton; in other words, a mathematical object employed in condensed matter physics to describe the collective behavior of a group of particles that can be treated as a single particle.

**Figure 5:**
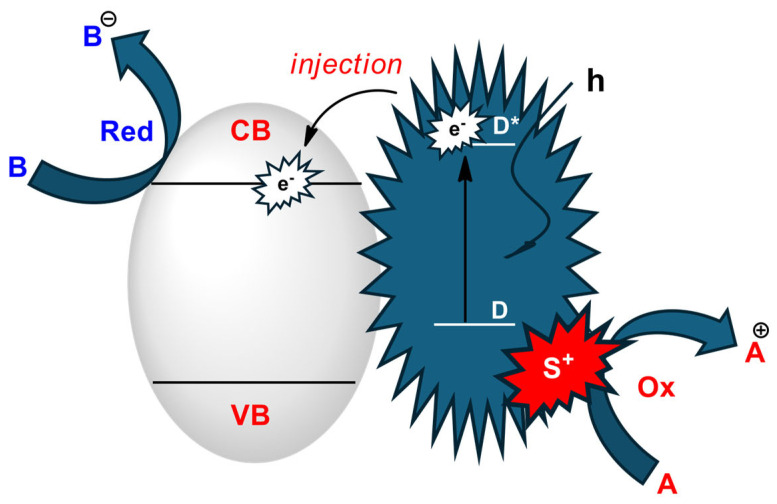
Mechanism of injection of the sensitizer into the semiconductor. * means excited state.

Some experimental evidence, such as the motion of particles inside materials, may be easily explained by using this model. In addition, semiconductors are materials with a high dielectric constant and, the higher it is, the more the charges (h^+^ and e^−^) are shielded from each other. This shielding lets the exciton dissociate into free charges that move toward the reactive sites on the surface of the semiconductor, in order to oxidize and reduce the substrates in terms of the hole h^+^ and excited electron e^−^, respectively [38]. Thus, in order to achieve efficiency in terms of redox catalysis, all steps in this process should be optimized, from pair generation to migration on the surface. Furthermore, recombination of the charge carriers should be avoided or, at least, limited. Immobilization and the consequent recombination of charges do not occur if the charges are efficiently separated. This process is strongly influenced by the dielectric constant and the quantum delocalization of the semiconductor. Generally, defects and doping are detrimental to the material acting as trap states and accelerate the recombination of the charges; however, catalysis can sometimes benefit from this morphological modification, even though the reasons are not well understood [39]. Semiconductors with a wide bandgap, such as TiO_2_ (3.2 eV), are of great interest because of their strong activity during photocatalytic reactions; however, this large bandgap requires irradiation with UV light, where absorption in the visible region is more important [40,41,42,43,44]. This can limit their application, but fortunately, several modification techniques were, hence, developed to tune light absorption. Properly functionalized molecules (named photosensitizers) can bind TiO_2_ through a strong or weak interaction (covalent bonding, hydrogen bonding, or electrostatic interaction), varying the photophysical properties of the resulting semiconductors; generally, shifting the absorption of the material toward a higher wavelength (bathochromic shift). Basically, the photon is absorbed by the photosensitizer, which is excited from ground state S to the excited state S*; at this stage, the electrons in the excited state are injected into the conduction band of the semiconductor. Formally, the photosensitizer results are oxidized after the injection of the electron, using S^+^, which is involved in substrate **A** oxidation; on the other side, substrate **B** is reduced at the interface with the semiconductor and the whole catalyst is restored; ready for taking part in another cycle (Figure 5) [45].

The photophysical characterization of semiconductors and modified semiconductors have both been deeply studied so far, and a brief description of them, employed in the examples reported in this review, is provided further in the text [46]. However, it is important to point out that organic reactions mediated by molecular photocatalysts are more widespread than those with heterogeneous analogues; this is likely due to the easier possibility of screening the reaction conditions during the optimization step [22]. Thus, the immobilization of privileged molecular photocatalysts onto an inert matrix is of great interest, hence it has been explored over the last decade [47]. In general, when the molecular photocatalyst is anchored via non-covalent interactions onto the inert matrix, and if this does not exhibit semiconductor behaviors (such as TiO_2_, BiVO_4_, etc.), its photophysical properties are generally unaltered from the homogeneous analogue and the mechanism previously explained, concerning dye-sensitized semiconductors, does not take place. There are some advantages to this approach; for example, functionalization occurs only on the surface, which is the area of the material that is accessible to the substrate and light, thus preventing the positioning of the photocatalyst in the bulk, where it is inactive. However, some disadvantages remain, mainly due to the decrease in the reaction kinetics of the catalyst that is no longer dispersed in solution, but this is a feature common to all immobilized catalysts [48].

## 3. Light Harvesting Improvement in the Flow Reactor

Generally, in order to reach high yields and selectivity in organic reactions, the mass and heat transport should be finely controlled; basically, both of them can be managed by convection, which in a standard batch process is controlled by stirring, but unfortunately this is limited by inhomogeneities [49]. For example, when dyes are mixed in vessels stirred either by bars or impellers, even small changes in geometry or the stirring rate can modify the mixing performance [50,51]. On the other hand, continuously flowing microreactors allow for rapid and homogeneous mixing because of their small dimensions. Microreactors can achieve complete mixing in microseconds, whereas classical reactors mix in a time scale of seconds or longer. However, the most important improvement due to performing a photo-promoted reaction in flow instead of batch, is the better irradiation of the solution thanks to the smaller channel dimensions of the reactor, compared to a batch reactor. The improvement in light harvesting in the solution is easily rationalized through the Lambert–Beer equation, described below.

$$\frac{I_{1}}{I_{0}}=e^{-k_{\lambda }l}$$

**Figure 6:**
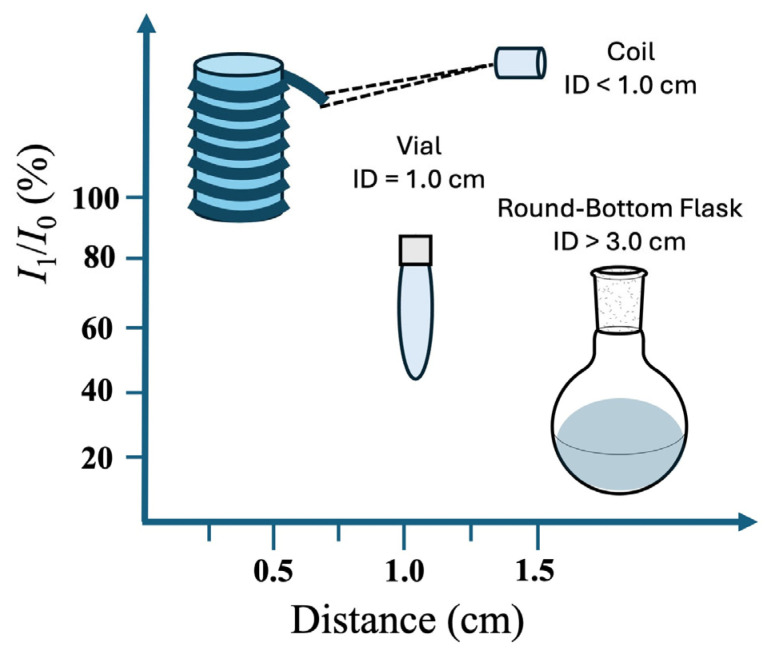
Attenuation of light with distance.

*I*_1_/*I*_0_ is the ratio between the transmitted and incident light, $k_{\lambda }$ is a constant that depends on both the concentration of the absorbing species and the wavelength, and l is the path length. From the equation above, the intensity of the transmitted light decreases as the path length increases. Thus, the absorbing species in the middle of the flask experience a lighter irradiation than the analogues close to the inner wall. Considering this, for the same volume, moving from the flask to the coil reactor, doubtless improves the efficiency of photocatalyzed reactions (Figure 6) [52].

This improvement is not only related to light-promoted reactions in a homogeneous medium, but even reactions mediated by heterogeneous photocatalysts can benefit. Packing tubular or coil reactors with heterogeneous photocatalysts allows them to be confined within thin transparent tubes, increasing the surface-to-volume ratio and permitting efficient and select irradiation [53].

## 4. Carbon Nitride

**Figure 7:**
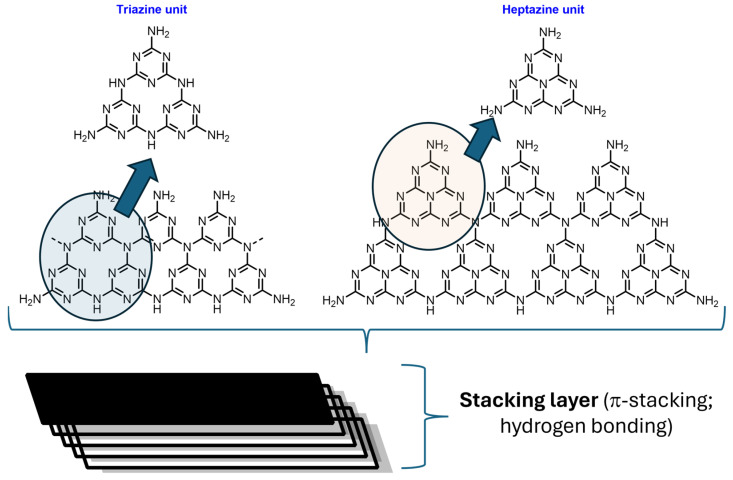
Triazine units and heptazine units are the building blocks composing the layers that, by stacking, make all the forms of g-CN.

Graphitic carbon nitride (g-C_3_N_4_, g-CN) is a polymeric semiconductor, which has attracted great attention so far; the main reason is that, whereas traditional TiO_2_ has no useful absorption within the visible spectrum, g-C_3_N_4_ has maximum absorption around 470 nm (blue), where the solar spectral irradiance is higher. Furthermore, g-C_3_N_4_ is simple to prepare through thermal polymerization of abundant nitrogen-rich precursors (mainly urea, cyanamide, and melamine). Even though this material has a long history, it was not until the beginning of the 20th century that carbon nitride attracted the attention of the scientific community for its application in water splitting, waste treatment, and synthesis. For detailed descriptions of the photophysical and morphological properties of g-CN, we recommend specific reviews on this material [54,55,56,57]; however, some basic aspects will be reported below. There are several allotropes of g-C_3_N_4_, and the units that compose the allotropes are mainly triazine and heptazine (Figure 7); its thermal stability is quite good, due to the aromaticity of the extended layer, with decomposition above 600 °C (TGA).

**Figure 8:**
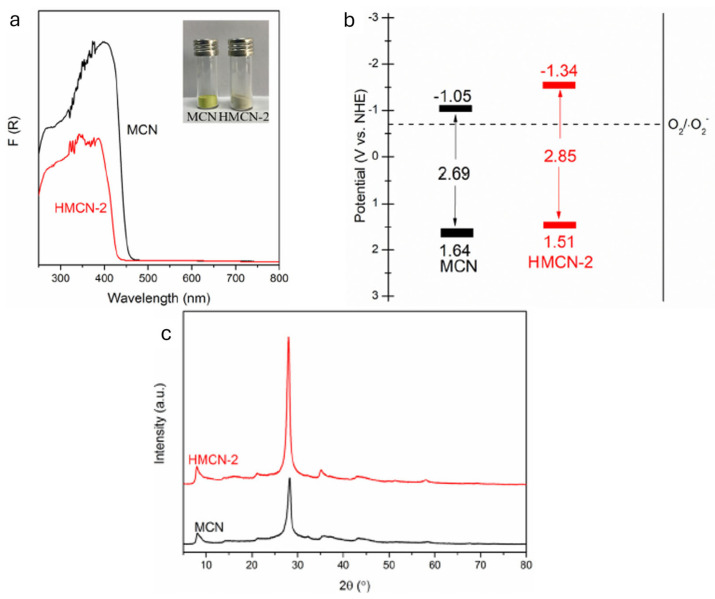
(**a**) Diffuse reflectance spectra of MCN and HMCN-2. (**b**) Band structure of MCN and HMCN-2. (**c**) XRD spectra of MCN and HMCN-2. (**a**–**c**) reprinted with permission from ref. [62]. Copyright 2021 Elsevier.

Its morphology and structure both influence the photocatalytic activity of g-CN, as described in the general section; in particular, it is important to highlight that the main disadvantages of g-CN concern its high recombination rate and its small surface area, which limits its reactivity due to the mass transfer of the reactant on the surface. A plethora of modifications have been employed to enlarge the specific surface area by controlling the morphology, by synthesizing nanostructured analogues, and by using hard templates, such as silica, for the synthesis of mesoporous analogues [58,59,60,61]. Furthermore, residual amine functionalities regarding g-CN have been exploited so far, to increase the yield and selectivity of the reactions of interest; in this regard, recently Wang and co-workers disclosed the efficient aerobic oxidation of alcohols to esters using acidified carbon nitride photocatalysts [62]. In this work, the acidified carbon nitride, named HMCN-2, was prepared by treating molten salt carbon nitride MCN with HCl solution. Physicochemical characterizations were performed to study the variations in the photochemical behavior and morphology, before testing the catalysts for oxidative coupling between benzyl alcohol and methanol. Basically, the treatment with acid did not modify the structure of the layered material, even though the sample exhibited sharper X-ray diffraction (XRD) peaks compared with MCN, thanks to the removal of the irregularity in the framework caused by K ions. The bandgap of the selected HMCN-2, calculated using Tauc plots, was 2.85 eV, a bit more than the untreated MCN (2.69 eV), and the color turned from yellow to white. Furthermore, the physicochemical measurements showed a clear shift in both the conduction band (CB) and valence band (VB) positions toward more negative potentials in HMCN-2 compared with MCN, this makes the reductive dioxygen activation easier, according to the speculation of the authors (Figure 8).

**Figure 9:**
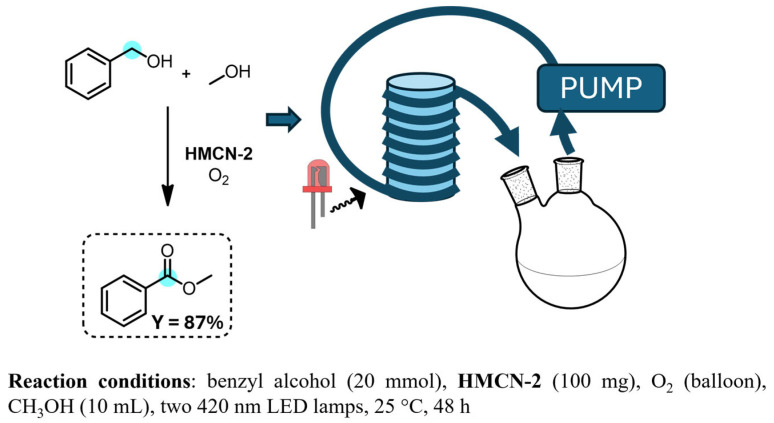
Oxidative coupling of benzyl alcohol with methanol promoted by HMCN-2 and light in continuous flow conditions.

However, selectivity toward the ester analogue is probably due to the acidic sites as well; in fact, the key intermediate, which was identified for the selective conversion toward methyl benzoate, was the hemiacetal, formed between methanol and benzaldehyde through acid catalysis, as demonstrated by the mechanistic test, employing the hemiacetal itself in the reaction condition, and via DFT calculations. The catalyst was tested on different substrates, showing good applicability on a wide range of substituted benzyl alcohol analogues. A gram-scale reaction was performed, both in batch and in continuous flow, by pumping the suspension with the catalyst and reagents, demonstrating that in the last case, the reaction outcome could be greatly improved, according to the benefits explained above, in the text on moving from batch to continuous flow conditions for photochemical reactions (Y_batch_ = 56% vs. Y_flow_ = 87%) (Figure 9).

**Figure 10:**
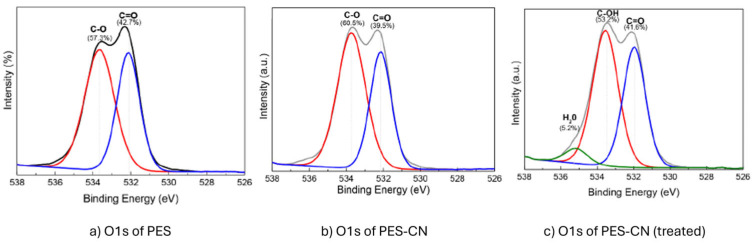
O1s XPS spectra of PES (**a**), PES-CN (**b**), and PES-CN (treated) (**c**). (**a**–**c**) reprinted with permission from ref. [63]. Copyright 2022 Elsevier.

Remaining on the topic of modified g-CN, using composites can be an approach to overcome some limitations to the use of g-CN itself. Recently, Silva et al. reported a work on the performance of a composite photocatalyst based on polyester-supported carbon nitride nanosheets, employed in the selective oxidation of anisyl alcohol [63]. Basically, PES (polyester fiber) was treated with a 1% *v*/*v* HNO_3_ solution to increase the hydrophilicity of the material, without modifying the framework. Concerning the photocatalyst, the g-CN underwent an additional thermal treatment to obtain the exfoliated material that was sonicated in UP water in several steps, before obtaining the desired photocatalyst. The resulting exfoliated photocatalyst was characterized using common experimental techniques, such as XRD and X-ray photoelectron spectroscopy (XPS). In particular, the XPS analysis showed a significant shift to higher energies in the O1s region in the exfoliated photocatalyst than in the untreated g-CN; this result suggests that the water molecules were chemisorbed onto the catalyst surface during sonication (Figure 10).

**Figure 11:**
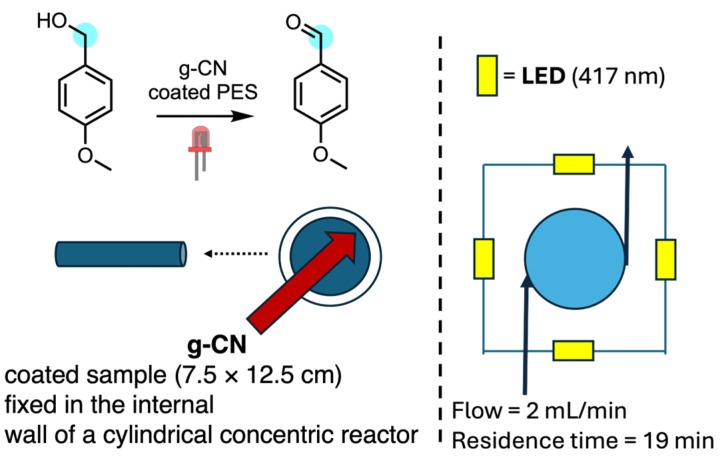
Set-up for the oxidation of anisyl alcohol into *p*-anisaldehyde, mediated by g-CN coated PES.

The authors speculated on this modification, affirming that hydroxyl groups on the exfoliated g-CN improved the link with the PES textiles. A test on the activity of the new synthesized catalyst was carried out, employing the oxidation of anisyl alcohol into p-anisaldehyde; the maximum yield of p-anisaldehyde was 85% after 30 min of irradiation of the reaction mixture employing the PES-modified photocatalyst with 1% *v*/*v* HNO_3_. The higher yield provided by this catalyst, compared to the other ones that were treated with different concentrations of HNO_3_, was attributed to the highest amount of photocatalyst on the PES surface, according to the UV–Vis and photoluminescence measurements. The improved loading of the photocatalyst onto the PES is due to the good wettability of the PES obtained after treatment, which improves the adhesion of g-CN on its surface. The photocatalytic oxidation of anisyl alcohol into p-anisaldehyde was investigated using the best performing composite (PES/g-CN) under the continuous mode of operation. Even though high stability was observed for reactions performed in continuous flow, the performances observed were generally slightly lower in terms of the yield than when performed in batch; in fact, the desired *p*-anisaldehyde was produced in amounts of 0.04 mM and 0.05 mM at residence times of 13 and 19 min, respectively, after 180 min (Figure 11).

**Figure 12:**
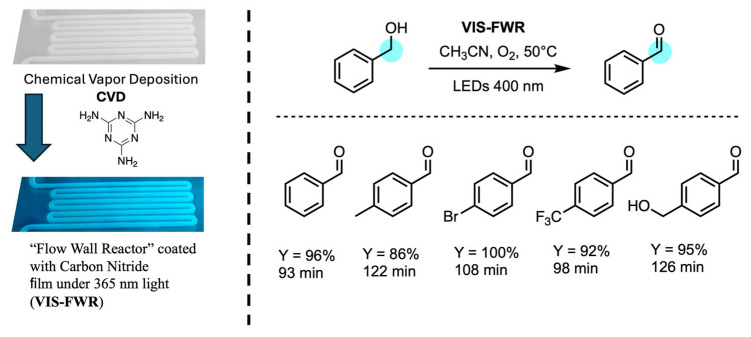
Oxidation of benzyl alcohol and scope extension involving a small range of benzyl alcohol derivates in continuous flow.

An interesting and innovative approach to employing g-CN as a photocatalyst in both batch and continuous flow conditions was disclosed by Giusto and Savateev [64]; in this work, the authors reported the preparation of reactors for photocatalysis through chemical vapor deposition (CVD) of g-CN onto the inner wall of vials and microfluidic apparatus for continuous flow. Here, the benefits of making a reactor with a supported catalyst for recycling, along with an improvement in the lifetimes of the photoactivated catalyst in its triplet excited state, were explored. The reactors were prepared according to the previous procedure disclosed by the same authors, employing melamine as a precursor [65]. CVD is a common technique used in the semiconductor industry to produce thin films. In this case, the glass of reactors is exposed to melamine that reacts on the surface to produce the desired deposit under a vacuum, then the low weight degradation side products are removed by the flowing nitrogen or argon. Oxidation of benzyl alcohol has been selected as benchmark reaction for the tests in batch, by employing the so-called “Visible Batch Wall Reactor” (Vis-BWR). A solution of benzyl alcohol in acetonitrile is irradiated at 420 nm for 24 h at 40° C in the presence of pure oxygen, resulting in the desired product with a yield of 87%. Then, the authors moved on to investigate the reaction in continuous flow; a microfluidic reactor with a channel diameter of 250 μm was coated with films of g-CN, by employing the CVD strategy to prepare the so-called “Visible Flow Wall Reactor” (Vis-FWR) (Figure 12).

**Figure 13:**
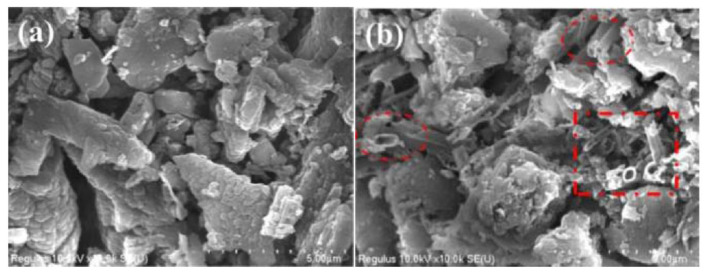
SEM image for g-CN (**a**) and 1.0 Ci-CN (**b**). In figure (**b**), the presence of a nanotubular structure is clear in square and cycle. (**a**,**b**) reprinted with permission from ref. [66]. Copyright 2023 American Chemical Society.

The microfluidic reactor was tested during the oxidation of benzyl alcohol; however, in this case, the main product was benzaldehyde, obtained in a much shorter reaction time then the analogous reaction in batch. Obviously, the much higher reactivity (96% yield in 93 min) is attributed to the higher surface area/volume ratio in the microchannels, which also prevents the consecutive oxidation of aldehyde to the corresponding acid. Morphological modifications can be conducted by adding additives during the synthesis of g-CN analogues. As reported above, morphological modification strongly changes the photochemical performance of the photocatalyst in a solid state. Recently, Guo and Xia disclosed the preparation of a range of spiro-imidazolidines via a four component one-pot methodology, employing a new variation of g-CN [66]. The best performing photocatalyst, named 1.0 Ci-C_3_N_4_, was synthesized through common thermal polymerization of melamine and glyoxal in a muffle furnace. The authors attribute the improvements in the photocatalytic performance to the partially distorted heptazine-based structure of the new synthesized g-CN analogue. In particular, glyoxal was employed to promote the insertion of C_2_ fragments into the heptazine framework, for varying the structure. The addition of 20% glyoxal to melamine provides g-CN materials with a nanotube structure, as demonstrated by the scanning electron microscopy (SEM) analysis (Figure 13).

**Figure 14:**
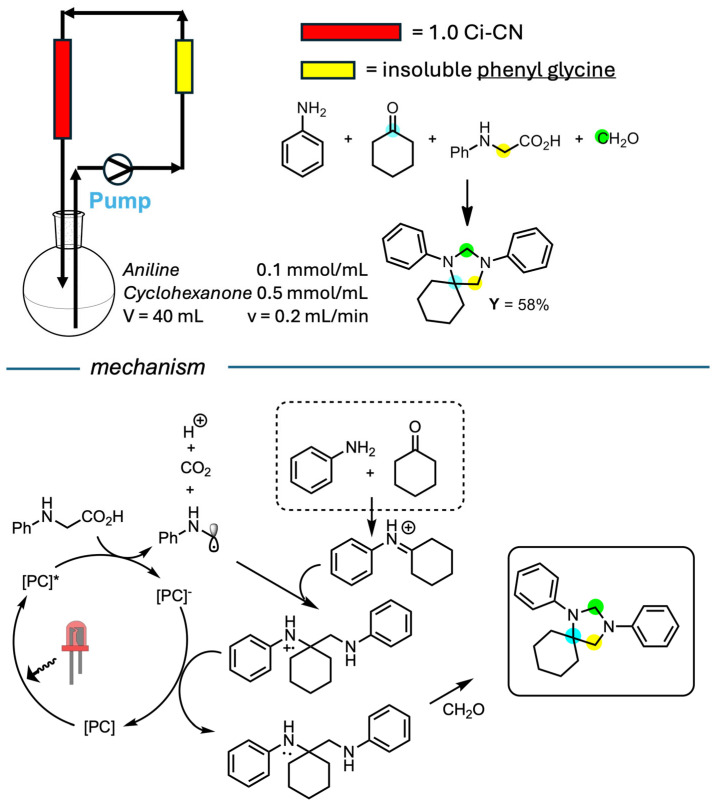
One-pot protocol mediated by 1.0 Ci-CN in continuous flow, in gram scale. * means excited state.

The wall thickness of the material also improves the specific surface area, increasing both the absorption of the light and the number of active sites. Furthermore, photoluminescence analysis shows that quenching of the new, reported catalyst is significantly lower than the standard bulk g-CN, indicating that the intercalation of the C_2_ fragment due to glyoxal could suppress the detrimental charge recombination. In terms of redox potential, the photophysical and electrochemical analysis (Mott−Schottky plots) show that the new, reported photocatalyst has a CB and VB of −1.19 V and 1.51 V, respectively. The 1.0 Ci g-CN was tested during the multicomponent reaction, employing aniline, cyclohexanone, N-phenylglycine, and paraformaldehyde for the synthesis of spiro-imidazolidines. In batch, the best results (80% yield) were obtained with light irradiation at 420 nm (blue LED) for 6 h. In this work, the authors performed the reaction in continuous flow for scaling up to grams; a small glassy tube of around 15 cm was packed with 200 mg of the heterogeneous photocatalyst and a solution of reactants (aniline, cyclohexanone, and paraformaldehyde) was pumped in the circulation line through a tube charged with N-phenylglycine, which is partially insoluble in DCM; working at a flow rate of 0.2 mL·min−1 with a residence time of 12 h, the desired product was collected in the amount of 0.68 g, with a yield of 58% (Figure 14).

**Figure 15:**
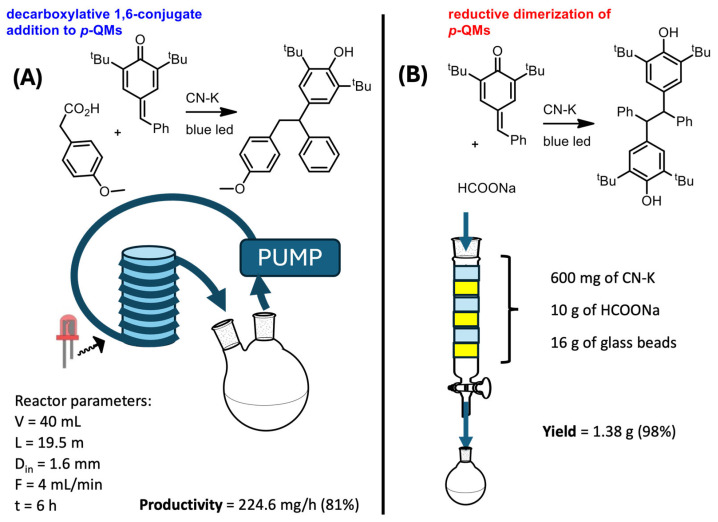

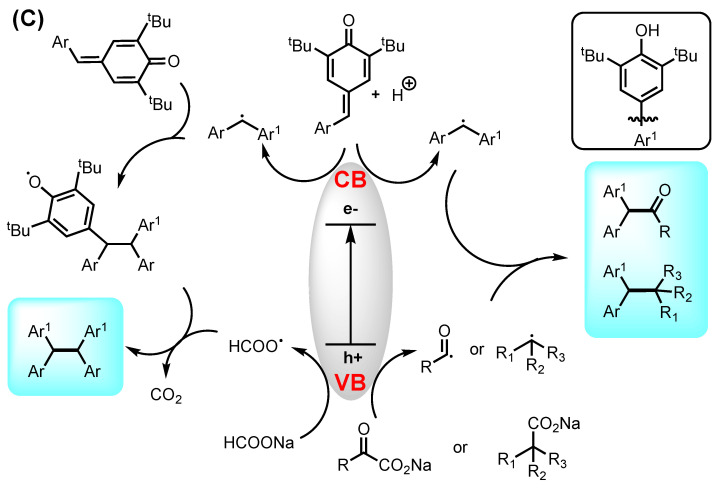
Continuous flow set-ups: (**A**) quasi-homogeneous decarboxylative 1,6-conjugate addition to *p*-QMs vs. (**B**) heterogeneous reductive coupling of *p*-QMs. (**C**) Proposed mechanisms.

The combination of structural modifications and doping can be a winning strategy to improve the performance of g-CN materials; in 2021, Tang and Cai reported on a work in this regard [67]. The authors developed a photocatalytic strategy for both decarboxylative radical addition to *p*-quinone methides (*p*-QMs) and reductive dimerization of *p*-QMs promoted by potassium-modified g-CN. A small range of g-CN materials doped with alkali metals was prepared via structure remodeling/doping of bulk g-CN, using the chlorides of the corresponding alkali metals. The new catalysts were tested in the photocatalytic decarboxylative 1,6-conjugate, with the addition of aliphatic carboxylic acids to *p*-QMs, selecting (4-methoxyphenyl)acetic acid as the model substrate. Surprisingly, the best performing doped photocatalyst, g-CN-K, was able to promote the reaction with a lower catalytic loading (0.25 mg/mL), even when compared to privileged molecular catalysts, such as [Ru(bpy)_3_]Cl_2_, *fac*-Ir(ppy)_3_, eosin Y, and 4CzIPN. XRD analysis, inductively coupled plasma (ICP) analysis, and transmission electron microscopy (TEM) imaging were performed to understand the main variations in respect to bulk g-CN and to try to rationalize the improvements. The TEM images show g-CN as an amorphous aggregate, whereas g-CN-K appears as a layered structure with nanometer-sized domains. The nanocrystallites present K ions intercalated to a poly(heptazine)-based framework. This morphological feature makes dispersion in the polar solvent easier, improving the optical absorption. Furthermore, the enhanced electron−hole separation which, as explained in the introduction to this review, strongly depends on the dielectric constant of the materials, was also confirmed by steady-state photoluminescence spectroscopy. The selected catalyst was employed in the dimerization of *p*-QMs as well; the corresponding tetraarylethane derivatives were obtained in nearly quantitative yields, employing sodium formate (HCOONa) as a reductant, and water instead of trifluoroethanol as a proton source. Even in this work, the continuous flow approach was finally exploited to scale-up the transformations. For the benchmark 1,6 addition to p-QMs, the reaction time increased from 3 to 36 h, moving to the gram scale; the authors overcame this problem by testing the reaction in quasi-homogeneous conditions (Figure 15A): by pumping the reaction mixture along with the heterogenous photocatalyst (dispersed in solution), the authors observed a productivity of 224.6 mg/h (six times faster than in batch gram scale). Similar results were observed for the dimerization of p-QMs performed in a heterogeneous fashion (Figure 15B), by employing a fixed-bed flow photoreactor made with glass beads, HCOONa, and the g-CN-K catalyst (98%, 1.38 g).

**Scheme 1:**
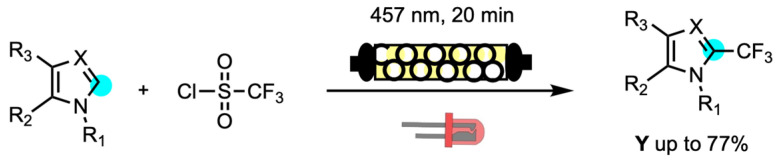
Trifluoromethylation in continuous flow, mediated by mpg-CN.

**Figure 16:**
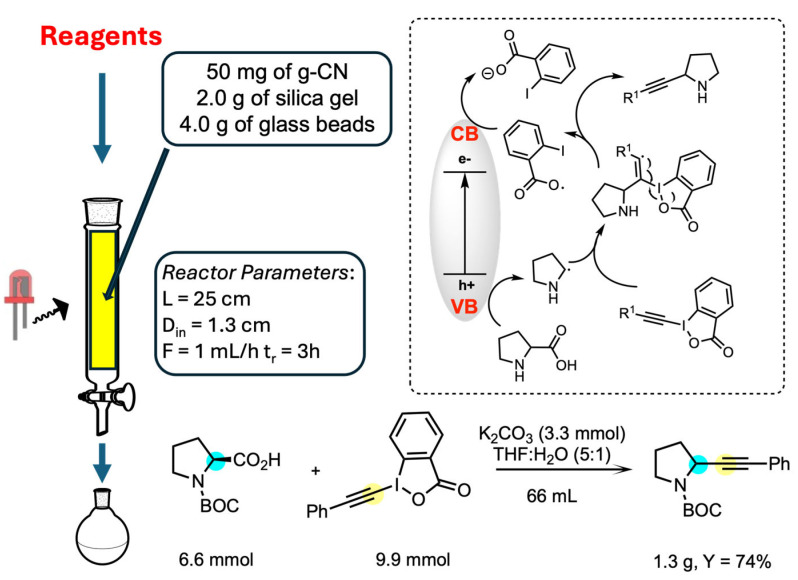
Continuous flow set-up for decarboxylative alkylation of BOC-protected proline.

Mesoporous materials have been deeply studied so far in all fields of heterogeneous catalysis, thanks to their greater surface area, which allows for a better interaction between the active sites of the catalyst and the reactants. In this context, mesoporous graphitic carbon nitride materials (mpg-CN) have been shown to boost reactivity compared to standard g-CN: their preparation was possible by employing hard templates during the calcination/polymerization steps. In this regard, in 2023, Vilé and co-workers disclosed a continuous flow trifluoromethylation of arenes and heteroarenes promoted by mpg-CN [68]. The trifluoromethylation of drug candidates is a pivotal topic in pharmaceutical chemistry, to increase lipophilicity and modulate bioavailability (Scheme 1). Thus, sustainable approaches that avoid the use of metals, which are concern for the pharmaceutical industry, are always welcomed. Mpg-CN has been prepared, according to the procedure disclosed by Reisner: [53] cyanamide was mixed with colloidal SiO_2_ (Ludox HS40) and water was slowly concentrated by heating the suspension at 70 °C. The resulting solid was calcinated at 550 °C, and the hard template was removed by treating the resulting solid with a solution of NH_4_HF_2_. A preliminary investigation was conducted in batch by varying the range of CN catalysts, which consisted of graphitic, nanolayered, and doped analogues; the best results, in terms of yield and selectivity, were obtained when employing mesoporous graphitic carbon nitride (96% conversion and 84% yield). Then, the reaction was optimized to be carried out in continuous flow; a transparent FEP tube was packed with mpg-CN, the inorganic base (K_2_HPO_4_), and glass beads. Even in this case, the continuous flow conditions allowed the optimal scale-up of the process for a prolonged time, with good productivity (Figure 16), which was almost twice that of the corresponding one in batch (0.62 mmol h^−1^ vs. 0.32 mmol h^−1^).

However, the design of new protocols and methodologies for the organic synthesis of standard graphitic carbon nitride (g-CN) remains a valid option. Recently, Wang and Zhang reported on the decarboxylative alkylation of BOC-protected proline with ethynylbenziodoxolone analogues promoted by light and g-CN [69]. The authors focused on reaction optimization in batch and on the study of the reaction mechanism. In addition, a brief recycling study was carried out and the reaction scale-up was investigated in flow. A simple column for silica gel chromatography was employed for this purpose, it was charged with 50 mg of g-CN, 2.0 g of silica gel to fill the interstitial spaces, and 4.0 g of glass beads; the mixture of reagents flowed through the column due to gravity, and 1.3 g of the product was collected with a yield of 74% (Figure 16).

The electrochemical characteristics of semiconductors could be of pivotal importance in some cases, and the redox potential, especially, cannot be neglected in this regard. For the reduction of recalcitrant substrates, a polymeric carbon nitride framework, namely potassium poly (heptazine imide) (K-PHI), showed interesting applications [70]. The standard protocol to prepare K-PHI consists of mechanochemical pretreatment of 5-aminotetrazole/LiCl/KCl eutectic; the resulting highly homogeneous mixture was then calcinated at 600 °C, resulting in highly crystalline K-PHI nanoparticles. The high-resolution transmission electron microscopy (HRTEM) and XRD analysis showed similarities with some zeolites, suggesting that potassium cations are in the cavities comprised of poly(heptazine imide) structures.

**Figure 17:**
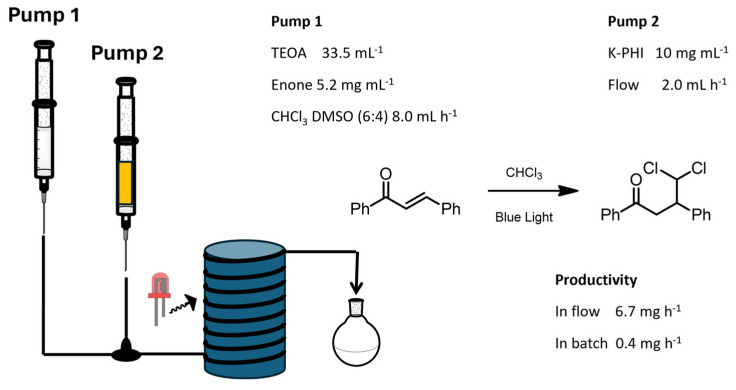
Schematic representation of the photoreactor set-up, with reaction parameters for the dichloromethylation of I-chalcone.

K-PHI was employed recently by Savateev and co-workers in the chloromethylation of enones [71]. TEOA (triethanolamine) was selected as the electron donor for the hole (hole scavenger); noteworthy improvements in terms of the reactivity were observed, in this case, in continuous flow, with a K-PHI suspension employed in a quasi-homogeneous flow regime. The overall productivity of the process in flow was 19 times higher compared to the batch conditions. As reported by the authors, stable quasi-homogeneous conditions in flow are possible due to the negative zeta potential (−40 mV) and size (100 nm) of the K-PHI nanoparticles, without using a gas–liquid system, thanks to electrostatic stabilization. Even in this case, moving from batch to flow conditions makes the reaction scale-up easier: pumping of the colloidal solution through the transparent FEP tube led to the collection of the desired product with a yield of 57% (Figure 17).

**Figure 18:**
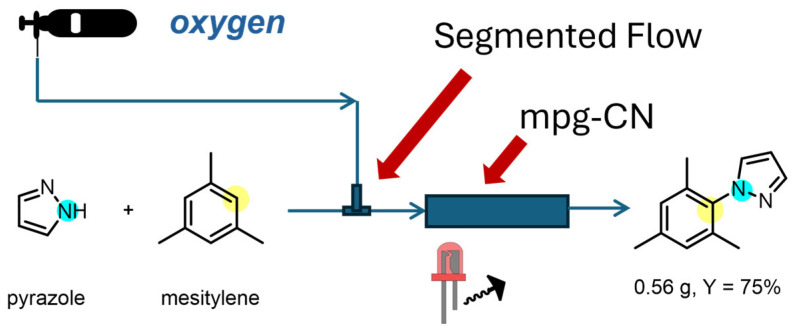
C-H azolation of arenes in continuous flow, with packed-bed reactor, promoted by mpg-CN.

At this point in the review, it appears evident that two strategies can be employed for handling solid catalysts in continuous flow, either suspension or immobilization inside the reactor. The latter strategy was successfully employed by Noël and co-workers in the photocatalytic C-H azolation of arenes, using mesoporous graphitic carbon nitride [72]. The heterogeneous photocatalyst was prepared according to the already reported procedure and tested with the oxidative addition of pyrazole and other nitrogen-based heteroaromatic cycles in regard to electron-rich arenes. Mesitylene was selected as a benchmark substrate and, after several optimization tests, the optimal condition was obtained in batch by employing K_2_S_2_O_8_ as an additive and pure oxygen as a terminal oxidant (80% yield after 15 h). The set-up in flow was obtained by packing a PFA tube with mpg-CN (Figure 18). A segmented flow was observed through the flow of both the solution of reagents and gas (oxygen); finally, the coil photoreactor was subjected to LED irradiation (365 nm), resulting in 0.56 g of the desired product, with a high turnover frequency (835.2 μmol g^−1^ h^−1^).

**Figure 19:**
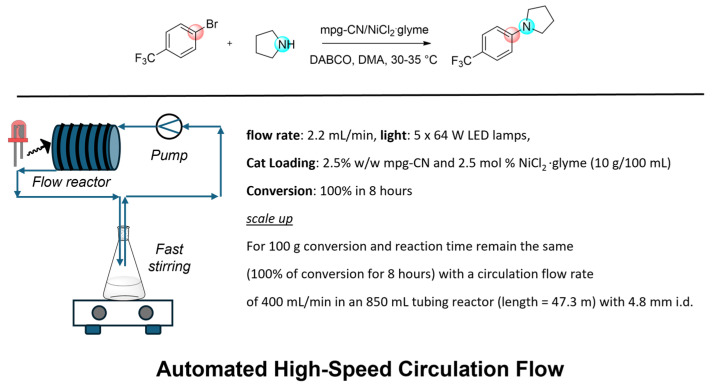
C–N coupling in an automated high-speed circulation flow, promoted by mpg-CN and NiCl_2._glyme.

Recently, Wu and coworkers reported on a practical and very efficient approach to carry out photocatalyzed reactions in continuous flow by employing mpg-CN [73]. The authors demonstrated that switching from continuous flow mode to a high-speed circulation flow mode can strongly enhance productivity, allowing the photocatalyzed reaction mediated by solid-phase catalysts to be quickly scaled up. The nickel/mesoporous graphitic carbon nitride (mpg-CN) blended catalyst was selected for the photo-mediated ligand-free Buchwald−Hartwig C−N coupling reaction. A capillary photoreactor packed with nickel/mpg-CN was tested, with scarce results in terms of conversion and yield (12% and 7%, respectively); thus, the authors moved to test their custom-designed circulation flow system and, after several tests to find the optimal conditions, they observed that with a flow rate of 2.2 mL/min, complete conversion into the desired product was obtained in 8 h, preventing aggregation and clogging in the C–N coupling between 1-bromo-4-(trifluoromethyl)benzene and pyrrolidine (Figure 19).

## 5. Polymeric Photocatalysts

This section is going to briefly cover the latest results reported in the literature on polymers that exhibit photochemical behaviors. The classification of materials involved in this regard is still linked to the structure and composition of the material itself. For example, covalent organic frameworks (COFs) are well-known materials that have found broad applications for gas separation and storage, due to the ease of the tunability of their porosity; this feature is strictly related to the synthetic conditions that can strongly control the crystallinity of the resulting product [74]. For these reasons, COFs are privileged materials for photochemical application; in fact, as already mentioned in the introduction to this review, both crystallinity and porosity affect the performance of the resulting heterogeneous photocatalyst. In respect to COFs designed for storage and separation, photoactive COFs present organic chromophores as monomers; these are linked to each other with a highly regular structure, which leads to long-range order. The incredible tunability, observed though varying the preparative conditions, has encouraged the exploration of these materials as metal-free semiconductors [75]. Briefly, the photophysical properties of a COF can readily be tuned by varying the organic precursors and/or by modifying its morphological features. However, this rule can be applied to each photoactive polymer; in fact, it is well known that organic functional groups that compose the polymer and its resulting morphology are both correlated with each other. This can be the case for materials that present extended π-conjugation, which leads to a microporous skeleton, if a hard template is employed in the preparation step.

**Figure 20:**
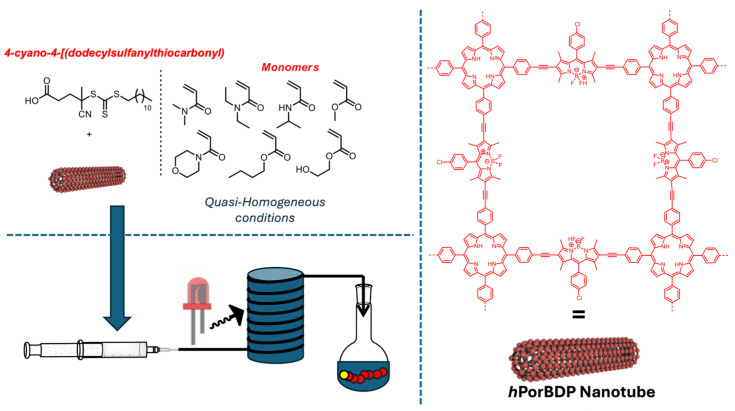
PET-RAFT polymerization in continuous flow, promoted by light and hPorBDP.

Unlike mpg-CN and CN analogues, the application of structural ordered polymers with optical features is still related to energy, and the reports in the literature are still scarce about organic methodologies [76]. However, their tunability has attracted interest in regard to its application for redox photocatalysis, and some reports have recently been published in this regard. In this section, we are going to critically discuss these works, highlighting the benefits to using these materials, and focusing on the related morphological features and shapes. Recently, Cai and co-workers reported on the synthesis of microporous nanocapsule-shaped photoactive polymers, named hPorBDP NTs [77]. The materials were prepared via Sonogashira–Hagihara cross-coupling polycondensation, using a tetra alkyne-terminated porphyrin (TEPP) and iodo-substituted diazaborane (BDP) as co-monomers. The capsule-like shape of the resulting polymeric photoactive material was obtained by employing silica nanorods as a hard template, with a length of 2.55 ± 0.25 μm and a diameter of 272 ± 32 nm, prepared using the standard hydrothermal process. After the cross-coupling polycondensation, performed by employing Pd(PPh_3_)_4_ and CuI in the presence of TEA as a base, the hard template was removed by treating the resulting encapsulated polymer, named SiO_2_@PorBDP NRs, with hydrofluoric acid (HF). The authors reported the full characterization of the polymer; in particular, solid state (SS) ^13^C NMR was performed to confirm the wholeness of the functional groups that represent the co-monomers after the treatment with hydrofluoric acid; furthermore, the microporous nature of the hPorBDP NTs (around 1.97 nm) was confirmed through the nitrogen adsorption/desorption isotherms at 77 K using the Brunauer–Emmett–Teller (BET) model. The XRD analysis showed the amorphous features of the hPorBDP NTs, whereas field emission scanning electron microscopy (FESEM) and TEM both showed the capsule-like shape with evidence of a hollow structure, when compared with the analogue before etching. The authors observed that hPorBDP NTs form a colloidal suspension in polar solvents, which is an important feature to work in continuous flow under quasi-homogeneous conditions. Before testing the catalyst in flow, some experiments in batch were performed: for this purpose, the photoinduced electron/energy transfer-reversible addition–fragmentation chain transfer (PET-RAFT) polymerization of N,N-dimethylacrylamide (DMA) was selected as a benchmark reaction, employing 4-cyano-4-[(dodecylsulfanylthiocarbonyl) sulfanyl]pentanoic acid (CDTPA) as a chain transfer agent. The mechanism involved in this reaction was the RAFT process, which operates via the degenerative chain transfer of radicals; in particular, CDTPA is activated by the photocatalyst, resulting in the radical initiator. The best results were obtained in continuous flow conditions, where well-defined homopolymers with a monomer conversion α > 75% and dispersities Đ < 1.15 were obtained with diverse monomer varieties, employing a ppm dosage of hPorBDP NTs; the flow setting consisted of pumping a solution of the catalyst, the initiator, and 4.0 M of the monomer at a flow rate of 15 μL·min^−1^ through a coil irradiated under yellow light (λ_max_ = 570 nm, 3.6 mW·cm^−2^), as a compromise for having narrow dispersity and a good yield (Figure 20).

Another interesting example of microcapsule-like shaped photoactive polymers, employed in synthesis, was reported by Tao and co-workers in 2021 [78]; they prepared a porous BODIPY-inspired polymeric microcapsule, where the shell of the hollow microcapsule was porous to facilitate the mass transfer of the reactant. The polymer was based on the MA-2IBDP monomer, which was prepared using the Friedel–Craft reaction between 2,4-dimethyl-pyrrole and p-hydroxybenzaldehyde, followed by esterification with methacryloyl chloride and iodination.

**Figure 21:**
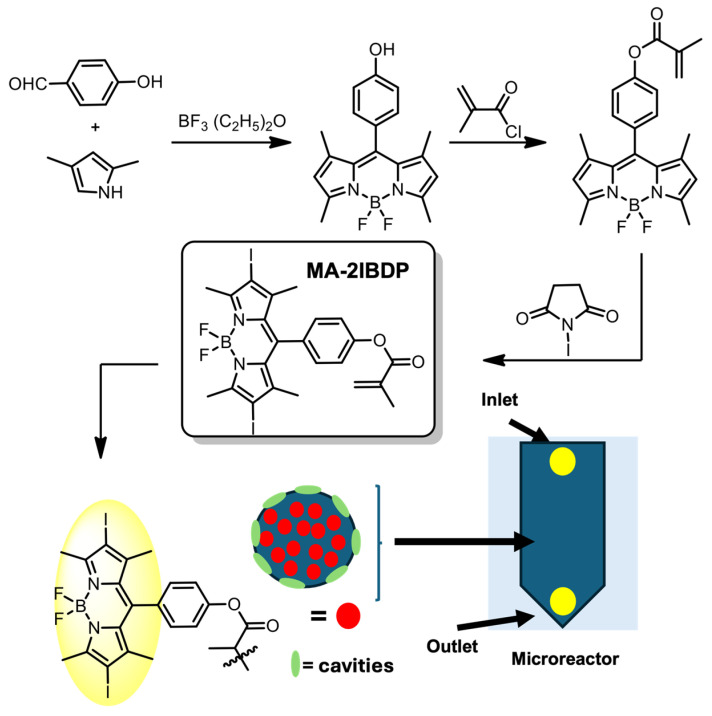
Schematic representation of MA-2IBDP-based microchip photoreactor.

The microcapsule-like photoactive polymer was prepared directly inside the microchip. In detail, the photoreactor was made by injecting a solution of Mowiol PVA-210 (Mw~67,000), glycidyl methacrylate, trimethylolpropane triacrylate, and 2-hydroxy-2-methylpropiophenone as the photo initiator, along with MA-2IBDP and 1-undecanol directly inside a microreactor (600 μm deep reaction tank, with a circular hole with a diameter of 1.7 mm for the inlet and outlet of the fluid), prepared by employing an EP-30-THG-S laser marking machine, operating at a wavelength of 355 nm, with a scanning rate of 600 mm s^−1^ and a current of 40 A (Figure 21).

Iodine on the BODIPY core was introduced to enhance the intersystem crossing (ISC), by populating the triplet state and improving the performance during photocatalysis. At this point, the properties of the triplet state of MA-2IBDP were evaluated, before and after the immobilization, via femto- and nano-second flash photolysis apparatus. Interestingly, the measures indicate that the ISC constant becomes seven times faster when MA-2IBDP is immobilized as a film, than in solution ($k_{ISC}=1/\tau_{S1\to T1}$, $3.7{\cdot 10}^{9}\;vs.\;2.7\cdot 10^{10}$ s^−1^). The synthesis of juglone via oxidation of 1,5-dihydroxynaphthalene (DHN) was selected as the benchmark reaction to evaluate the performance of the prepared catalyst. First, the reaction was performed in homogenous conditions employing MA-2IBDP (light irradiation was performed via a Xe lamp, with a 400 nm filter, operating at a power of 20 mW cm^−2^); after 1 h, the reaction reached the equilibrium and a yield of 88% was observed. The authors prepared several porous microcapsules with different loadings of MA-2IBDP (0.03, 0.07, 0.14, 0.28, and 0.70 wt%) and their photocatalytic abilities were evaluated in flow.

Working with a flow rate of 3 mL h^−1^, a yield of 88% at the equilibrium was observed, employing the photoreactor prepared with a loading of 0.07 wt% (which is comparable to the homogeneous one, where 3 mL of the reaction mixture took 1 h to reach the chemical equilibrium); working with a photoreactor, which presents a higher loading of the catalyst (0.7 wt%), the rate constant is 10 times its homogeneous counterpart.

It is noteworthy that the authors successfully tested the photoreactors using the aza-Henry reaction, the Alder-ene reaction, and the oxidation of thiols to disulfides as well, observing a high conversion rate (>95%).

**Figure 22:**
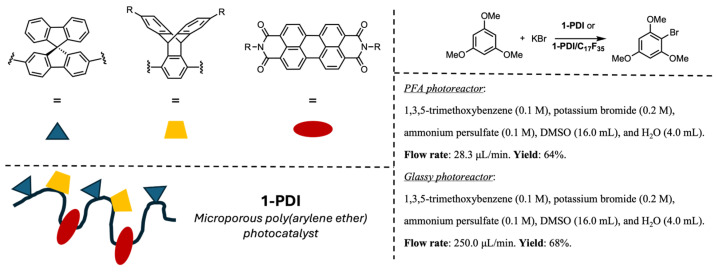
Bromination of 1,3,5-trimethoxybenzene promoted by 1-PDI, supported on both PFA tubes and glass.

In 2022, Swager and coworkers reported on a promising protocol to prepare a new class of photoactive organic polymers. These new materials combine the advantage of using tiny molecular dyes with the recyclability of heterogeneous catalysts [79]. Moreover, *t*-bu-triptycene hydroquinone, spirobifluorene dibromide, and the photo-activated dye, perylene diimide PDI, were employed as co-monomers in a polycondensation process, resulting in a deep red microporous poly(arylene) ether 1-PDI. This material was mainly characterized by a N_2_ adsorption isotherm, which showed microporous features, and displayed absorption and steady-state photoluminescence spectra. Several photocatalyzed reactions were studied in batch, such as the aerobic oxidation of thioether to sulfoxide, the dehalogenative reduction of aryl bromide atom-transfer radical addition to alkenes, and the anti-Markovnikov hydroetherification of diaryl akenes. Continuous flow reactions were performed by both employing a photoreactor prepared by slowly evaporating a solution of 1-PDI in THF through the deposition of a thin film layer, and by preparing a 1-PDI analogue with a relatively high fluorine content to deposit it onto a narrow inner wall of the PFA tubing. Both photoreactors were tested during the bromination of 1,3,5-trimethoxybenzene (Figure 22).

## 6. Molecular Photocatalysts Supported on Inert Matrixes

**Figure 23:**
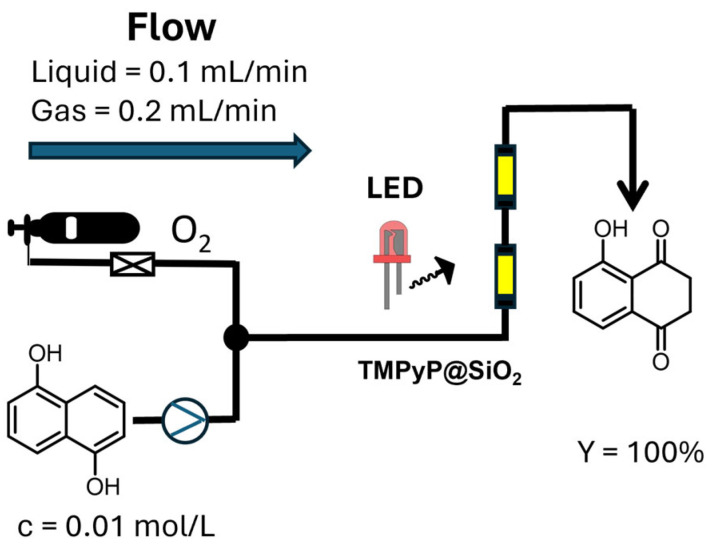
Schematic representation of set-up employed in the oxidation of DHN in continuous flow by TMPyP@SiO_2_.

The strategy to prepare heterogeneous photocatalysts described in this section is inspired by the well-known approach employed to anchor soluble catalysts onto inert matrixes [80]. Immobilization proceeds by bounding the molecular catalyst, which can be previously modified to install functional groups that are complementary to the functional groups on the matrix. It is noteworthy that molecular photocatalysts, anchored onto inert supports, show a lower fluorescence quantum yield in the solid state compared to its homogeneous counterpart. Fluorescence quenching can be typically observed when a fluorophore is attached onto a solid particle, due to the intermolecular energy transfer or inner filter effects present in heterogeneous conditions [81]. The main characteristics of the support should be high mechanical and chemical stability and tolerance to working conditions, inertness toward the reagents and products, sustainability, and recyclability. Polystyrenes [82], silica [83], and glassy materials are all privileged matrixes in this regard, because of their well-known, deeply disclosed properties [84,85,86,87]; furthermore, anchoring can be performed through different strategies, such as covalent bonding, electrostatic interaction, affinity, and cumulative weak interactions (such as π-stacking and London dispersion forces). For example, the negative charges on silica materials can be employed to anchor cationic photocatalysts through electrostatic interactions, as demonstrated by Amara and co-workers in 2020 [88]. Both tris(bipyridine)ruthenium(II) [Ru-(bpy)_3_^2+^] and the sulfonate salt of 5,10,15,20-tetrakis(N-methylpyridinium-4-yl)porphyrin (TMPyP^4+^) were anchored onto silica, by mixing the cationic photocatalyst (PC) with commercial silica (SiO_2_) in water. Then, the resulting colored powder was filtered and washed several times with fresh water. First, the authors performed a test in batch, to evaluate the performance of the catalyst compared with the homogeneous analogue. Even in this case, the oxidation of 1,5-dihydroxynaphthalene (DHN) into juglone was selected as the benchmark reaction. Acetonitrile was selected as a solvent due to its long ^1^O_2_ lifetime (81 μs). Experiments were carried out both in homogeneous and heterogeneous conditions; even though the reactivity decreased slightly when moving from homogeneous to heterogeneous conditions, the best results were observed using porphyrin as a photocatalyst. Thus, the authors moved to investigate the reaction in continuous flow, employing a photoreactor made by feeding a transparent tube with the PC@SiO_2_ catalyst (1.3 g per cartrid¼ 1/4″ PTFE tubing, volume = 1.5 mL). The resulting reactor was irradiated with LED strips (white). The substrate was pumped through a T-mixer, along with air, directly into the photoreactor; the best results (yield 100%) were obtained employing a loading of 4.5 mg·g^−1^ of TMPyP@SiO_2_, a concentration of 0.01 (mol L^−1^) of the starting material, a flow of liquid and gas of 0.1 mL min^−1^ and 0.2 mL min^−1^, respectively, and two cartridges (Figure 23).

**Figure 24:**
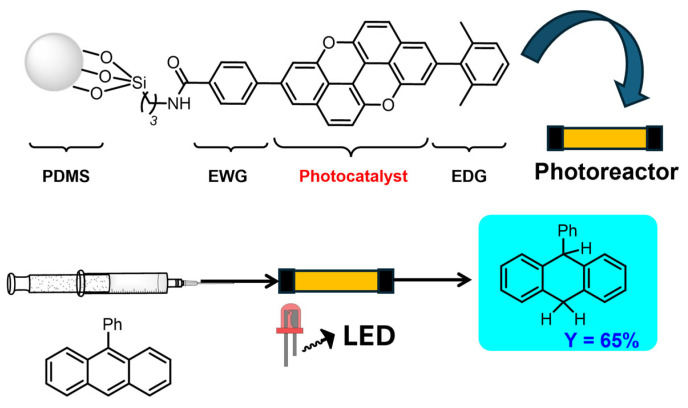
Birch reduction of phenyl naphthalene by PXX-PDMS in continuous flow.

Concerning the silicon-based support, recently, Bonifazi and co-workers reported the photoreduction of anthracenes mediated by peri-xanthenoxanthene to afford the Birch adduct, where the reaction was tested in continuous flow by supporting the photocatalyst PXX onto polydimethylsiloxane (PDMS) microparticles [89]. Anthracene was selected as the benchmark substrate due to its affordable reduction potential (−2.0 V vs. SCE). First, the reaction conditions were optimized in homogeneous conditions mainly by varying the solvent, the base, and the light longwave. The desired product was obtained with a yield of 84%, employing DMSO as a solvent, DIPEA as a sacrificial reductant, and the LED operating at λ = 405 nm. Interestingly, working in homogeneous conditions requires a very diluted concentration of the catalyst (around 2% *w*/*v* or less) to avoid self-aggregation. The authors investigated the scope extension and studied the mechanism of reaction; thus, to explore the potential of the catalyst, from a more sustainable point of view, they moved to prepare the heterogeneous analogue and test it in the benchmark reaction. Polydimethylsiloxane (PDMS) beads were selected as the support for this transformation [90]. A slightly modified photocatalyst with a (3-aminopropyl)triethoxysilane moiety was prepared to enable grafting onto the PDMS beads, whereas the xylyl substituent was installed to increase the solubility for the grafting. The two different natures of the two substituents, one electron poor (EWG) and one electro rich (EDG), counterbalance their electronic effect, resulting in a modified photocatalyst, with almost the same photo-electro characteristics as the original one. The grafting was performed in THF at room temperature in the presence of triethylamine. The resulting PXX-PDMS was tested in batch, employing 9-phenyl anthracene as the starting material, the yield (68%) was slightly lower than the analogue in homogeneous conditions; furthermore, the stability of the new catalyst was evaluated, demonstrating that the catalyst was stable over at least five runs. Finally, the photoreactor was prepared by pumping a suspension of PXX-PDMS (2.5% *w*/*v*) in EtOH through a glass tube with an internal diameter of 0.15 mm, equipped with a glass frit. Yields comparable (around 65%) to the heterogeneous counterpart were obtained with flow rate of 0.6 mL/h and by the flow of a solution of the starting material with a concentration of 0.02 M in DMSO. Concerning the conventional supports for photocatalyst immobilization, polystyrene materials play a pivotal role (Figure 24).

**Figure 25:**
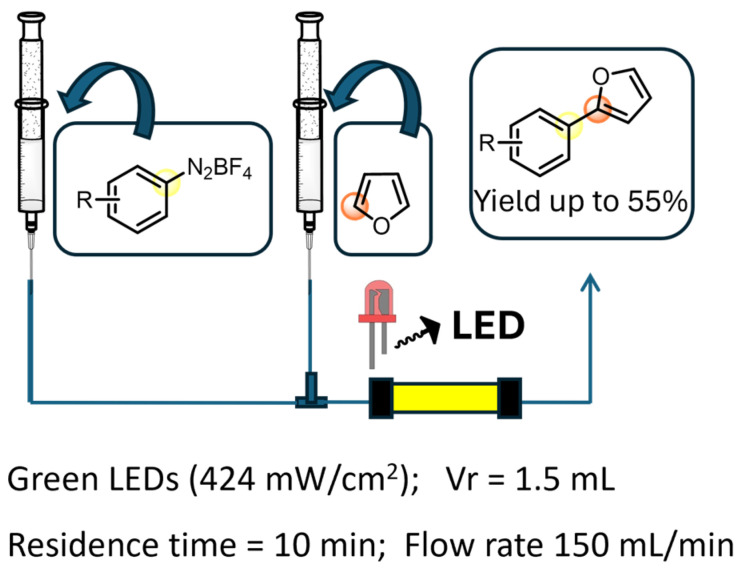
Schematic set-up of coupling between benzenediazonium salts and furan, promoted by MR-EY, in continuous flow.

In 2022, Puglisi and co-workers published a work in this regard, by testing eosin Y, supported on Merrifield’s resin, in the coupling of aryl diazonium salts with furans [91]. High-loading Merrifield resin was selected for this purpose (1.2 mmol/g, 100–200 mesh); the strategy of immobilization exploited the nucleophilic substitution of eosin Y, deprotonated by DIPEA, onto the methylene chloride group of the Merrifield resin, affording the resulting MR-EY heterogeneous photocatalyst with a loading of 0.168 mmol/g. Thus, the authors tested the photocatalyst in batch, employing a homemade photoreactor equipped with LEDs operating at 530 nm (green light), which is the maximum absorbance range for eosin Y. The best condition was found by using p-chlorophenyl diazonium salt and furan, with a stoichiometric ratio 1:20 and 2 mol% of the photocatalyst; in these conditions, the desired product was collected with a yield of 75% after 2 h. For the continuous flow experiments, 1.23 g of MR-EY (0.207 mmol of EY) was charged, along with 37 glass balls (5 mm), inside a 100 mm × 10 mm L × ID glassy column. The reactor was surrounded by three cylinders equipped with green LED strips (424 mW/cm^2^); two solutions, aryldiazonium salt in DMSO and furan in DMSO, were pumped using a syringe into the reactor through a T-mixer, with a certain flow rate, to obtain a residence time of 40 min and maintain an overall flow rate of 38 μL/min (62% yield, Figure 25).

**Figure 26:**
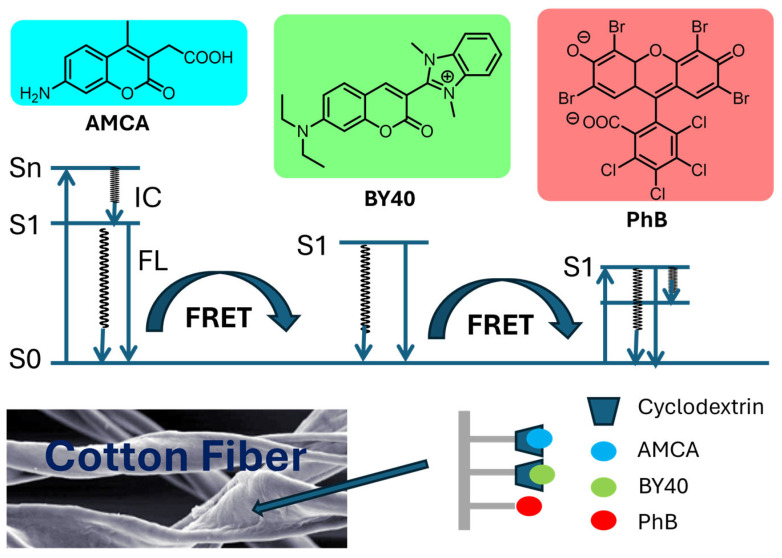
Light-harvesting system AMCA−BY40−PhB cotton fiber.

Alternative and sustainable inert matrixes have been investigated so far as support for molecular dyes and photocatalysts. Cotton fiber was exploited as support for building an efficient and heterogeneous light-harvesting system (LHS) by Zhao and co-workers in 2023 [92]. LHSs are molecular clusters composed of several photoactive compounds inspired by photosynthesis and, in this work, the system was composed of the amino-methyl coumarin acid AMCA, C.I. Basic Yellow 40 BY 40, and phloxine B PhB. PhB is the photocatalyst involved in the activation of singlet oxygen ^1^O_2_ through energy transfer; unfortunately, PhB only absorbs green light (500–570 nm), resulting in poor light utilization (if we look at the valorization of sunlight for organic chemical reactions); however, the authors performed a preliminary fluorescence analysis, in which they observed an overlap between the emission spectrum of BY 40 and the absorption spectrum of PhB, which presupposed the possibility of a FRET process. FRET (Förster resonance energy transfer) is a physical phenomenon in which a photoactive compound, named antenna, is involved in decay from an excited state, reached after the absorption of a photon; the released energy is captured by another photoactive compound, which reaches the excited state without formally absorbing the photon from the light source itself. In this work, the authors identified a system which involves two FRET processes, consisting of the photoactive compounds described above (AMCA, BY 40, and PhB). Both AMCA and BY 40 were anchored onto cationized cotton fiber as sulfobutyl-β-cyclodextrin (β-SCD) inclusion complexes to avoid aggregation, whereas PhB was anchored without further modification. For this purpose, cotton fiber (MCF) was cationized by employing 3-chloro-2-hydroxypropyltrimethyl ammonium chloride (CHPTAC) in an alkaline solution; the degree of cationization, and amount of the three photocatalysts, were optimized to enhance their uptake onto the inert matrix (Figure 26).

After several photochemical measurements, the resulting heterogeneous system was tested according to the cross-dehydrogenative coupling (CDC) reaction between N-phenyl-tetrahydroquinoline (N-phenyl-THIQ) and nitromethane in a methanol aqueous solution (MeOH/H_2_O = 1:1). The aza-Henry adduct was collected with a yield of 99.08% in 8 h, employing AMCA/BY 40/β-SCD-PhB-MCFs. Thus, after exploring the substrate scope and the mechanism, the authors tested the catalyst in continuous flow conditions: the desired product was collected with a yield of 93% by pumping a solution of N-phenyl-THIQ (24 mM), with a flow rate of 12 mL/min in 3.5 h.

## 7. Conclusions and Perspectives

In this review, we discussed, from a critical point of view, the latest works disclosed on photo redox catalysis and energy transfer catalysis, in synthetic transformations, achieved in continuous flow reactors, by heterogeneous photocatalysts. After focusing, in the introduction, on the possible advantages that can be gained by moving from batch to flow conditions in photocatalysis, the works were discussed according to the type of materials that compose the heterogenous photocatalysts. Particular attention was placed on the performance of the catalysts, compared with batch processes, where possible. It is important to point out that some of the studied reactions are of interest for the valorization and functionalization of bulk molecules, such as the oxidation of alcohol into aldehydes and carboxylic acids by atmospheric oxygen; on the other hand, reactions of interest for the fine chemical industry, such as C–C and C–X cross-coupling reactions, have been reported as well.

We believe that both high recyclability and a decrease in energy-demanding processes enabled by heterogeneous photocatalysis in continuous flow will lead to even more publications on this topic, with a perspective towards large-scale photosynthetic methodologies for industrial purposes. Furthermore, the possibility to work in quasi-homogeneous conditions, employing a new generation of materials paves the way to protocols in which solid-phase photocatalysts can be employed with reactivities comparable to their homogeneous counterparts.

## Data Availability

No new data were created or analyzed in this study. Data sharing is not applicable to this article.
